# The Transition Between Slow-Wave Sleep and REM Sleep Constitutes an Independent Sleep Stage Organized by Cholinergic Mechanisms in the Rostrodorsal Pontine Tegmentum

**DOI:** 10.3389/fnins.2019.00748

**Published:** 2019-07-23

**Authors:** Carlos Carrera-Cañas, Miguel Garzón, Isabel de Andrés

**Affiliations:** Departamento de Anatomía, Histología y Neurociencia, Facultad de Medicina, Universidad Autónoma de Madrid, Madrid, Spain

**Keywords:** intermediate state, transition state, perilocus coeruleus α, REM sleep, slow-wave sleep, carbachol, polygraphic recordings, cat

## Abstract

There is little information on either the transition state occurring between slow-wave sleep (SWS) and rapid eye movement (REM) sleep, as well as about its neurobiological bases. This transition state, which is known as the intermediate state (IS), is well-defined in rats but poorly characterized in cats. Previous studies in our laboratory demonstrated that cholinergic stimulation of the perilocus coeruleus α nucleus (PLCα) in the pontine tegmentum of cats induced two states: wakefulness with muscle atonia and a *state of* dissociated sleep we have called the SPGO state. The SPGO state has characteristics in common with the IS, such including the presence of ponto-geniculo-occipital waves (PGO) and EEG synchronization with δ wave reduction. Therefore, the aims of the present study were (1) to characterize the IS in the cat and, (2), to study the analogy between the SPGO and the different sleep stages showing PGO activity, including the IS. Polygraphic recordings of 10 cats were used. In seven cats carbachol microinjections (20–30 nL, 0.01–0.1 M) were delivered in the PLCα. In the different states, PGO waves were analyzed and power spectra obtained for the δ, θ, α, and β bands of the EEG from the frontal and occipital cortices, and for the θ hippocampal band. Statistical comparisons were made between the values obtained from the different states. The results indicate that the IS constitutes a state with characteristics that are distinct from both the preceding SWS and the following REM sleep, and that SPGO presents a high analogy with the IS. Therefore, the SPGO state induced by administering carbachol in the PLCα nucleus seems to be an expression of the physiological IS of the cat. Consequently, we propose that the PLCα region, besides being involved in the mechanisms of muscle atonia, may also be responsible for organizing the transition from SWS to REM sleep.

## Introduction

Multiple transitions occur between the different stages of the sleep wakefulness cycle (SWC) but these transitions are not so well-characterized as the SWC stages themselves. Transition states are difficult to analyze because of their short duration and complexity. However, these periods are critical since they involve changes in multiple brain areas that are essential for the proper establishment of each sleep stage (Durán et al., 2018; Sánchez-López et al., 2018). One of these critical transition states is the slow-wave sleep (SWS) to rapid eye movement (REM) sleep transition.

In cats, pontogeniculooccipital (PGO) activity is not only associated to REM sleep, it also appears about 1 min before REM sleep onset during SWS; therefore, the SWS with PGOs was considered the transitional period in this species (Ursin and Sterman, 1981). Pioneer studies in the rat had defined a transition state of shorter duration (1 to 5 s) that preceded REM sleep called the intermediate state (IS) (Gottesmann, 1973). The IS had large-amplitude sleep spindles in the frontal cortex electroencephalogram (EEG) and low frequency θ activity in the dorsal hippocampus (Gottesmann, 1973). The IS was also studied in the cat where it was found to share the same EEG characteristics of the rat IS, with the addition that PGO waves could also be observed (Gottesmann et al., 1984). The latter authors also reported that the IS was shorter in cats than in rats (1 to 3 s), only present in 30% of the animals, and one or more IS episodes could be observed just before and occasionally even after REM sleep. Therefore, for those authors the cat IS was not the whole SWS interval preceding REM sleep in which PGO activity was already present. In relation with IS mechanisms, it was reported that the forebrain would be transiently disconnected from the brainstem during IS in cats and rats (User et al., 1980; Gottesmann and Gandolfo, 1986; Piallat and Gottesmann, 1995; Gottesmann, 1996).

Recently the IS has again attracted interest, particularly the temporal sequence of the events that occur within this stage. On the one hand, rat studies on the dynamics of sleep stages in different brain structures, such as the neocortex and the hippocampus, have indicated that there may be functional dissociations between these structures and it seems that the sleep state showing the least concordance among the different brain structures is the IS (Durán et al., 2018). According to the latter authors, IS, which would have an average duration of 18 s, is identified by a decrease in δ activity accompanied by increased θ activity and the presence of sleep spindles, although these activities may not be simultaneous in the different cortical regions. On the other hand, high frequency oscillations (HFO, 110–160 Hz), which are modulated by θ rhythm and modified by behavioral state (Scheffzük et al., 2011; Cavelli et al., 2018), have been reported to completely delimit the IS in the rat since they are minimal during SWS and maximum during REM sleep (Sánchez-López et al., 2018). Therefore, a new definition for the IS has been proposed in the rat. It would be a period of approximately 20 s during which electroencephalographic variations occur before motor changes. In relation with IS mechanisms, structures located in the dorsal oral pontine tegmentum, like the sublaterodorsal nucleus (SLD) of the rat, have been proposed as being responsible for the coordination of all these events taking place during IS (Sánchez-López et al., 2018). In fact, REM sleep is enhanced after SLD glutamatergic stimulation or GABAergic disinhibition of this region, however, SLD cholinergic stimulation of this region produces wakefulness (W) with muscle atonia (Boissard et al., 2002).

The perilocus coeruleus α nucleus (PLCα) in the rostrodorsal pontine tegmentum of humans and cats or its homolog in rats, the SLD, is a part of the locus coeruleus complex (LCC) together with the locus coeruleus α (LCα) and the subcoeruleus (SCoe) nuclei (Figure 5). These regions contain cholinergic, noradrenergic and serotonergic neurons (Sakai et al., 2001; Reinoso-Suárez et al., 2011), as well as glutamatergic and GABAergic neurons (Boissard et al., 2002; Lu et al., 2006). The PLCα shows active neurons during REM sleep (PS-on neurons, Sakai et al., 2001) and injury or inhibition of that region can decrease or abolish REM sleep (Crochet and Sakai, 1999). Besides, the PLCα was initially considered as the region responsible for cholinergic REM sleep generation (Sakai, 1988; Vanni-Mercier et al., 1989). However, pioneer studies in the cat had indicated that cholinergic stimulation of the dorsal pontine tegmentum produced W without muscle tone (Van Dongen et al., 1978). Later studies with carbachol microinjections in the dorsal oral pontine tegmentum specifically located in the PLCα confirmed these results (Reinoso-Suárez et al., 1994), in contrast to the clear triggering of REM sleep produced by cholinergic stimulation of the near ventral part of the oral pontine reticular nucleus (vRPO, Reinoso-Suárez et al., 1994; Garzón et al., 1997, 1998). A more recent study (Moreno-Balandrán et al., 2008) has shown that small-volume low doses of carbachol (0.01–0.1 M) in the PLCα produce W with atonia alternating with another state in which the cat shows behavioral sleep, but shows a polygraphic pattern that does not conform to the typical characteristics of the spontaneous stages of SWS or REM sleep, although it has features common to both. Like physiological REM sleep, the sleep induced by cholinergic stimulation of the PLCα nucleus presents muscle atonia and PGO activity. Nevertheless, the EEG presents a striking synchronization but the typical δ activity of SWS is reduced while θ and α activity is increased. In addition, the hippocampal EEG also shows θ activity that is less rhythmic than that during spontaneous REM sleep (Moreno-Balandrán et al., 2008). These features of carbachol PLCα-induced sleep are reminiscent of the characteristics of IS in the transition between SWS and REM sleep (User et al., 1980; Gottesmann et al., 1984; Gottesmann and Gandolfo, 1986; Piallat and Gottesmann, 1995; Gottesmann, 1996; Durán et al., 2018; Sánchez-López et al., 2018). In order to establish the functional significance of PLCα in relation to the SWC mechanisms, these observations led us to further analyze the effects of low carbachol doses in this region, investigating their relationship with physiological SWS and REM sleep but also with the IS. Additionally, in light of the scant existing knowledge on cat IS (Gottesmann et al., 1984), we have also tried to further characterize the bioelectric features of IS in this species so as to compare it with carbachol-PLCα induced sleep.

## Materials and Methods

### Subjects and Surgery

We used 10 adult cats (nine males and one female). All experiments were carried out in accordance with the European Community Council Directive (2010/63/UE) and approved by the Institutional Animal Care and Use Committee of the Universidad Autónoma of Madrid (Spain) and the competent regional government agency (PROEX 004/15). Surgery was performed under general anesthesia [Medetomidina (Domtor) 0.1 mg/kg i.m. and Pentobarbital (Dolethal) 14 mg/kg i.p. using aseptic techniques. Using the coordinates of the Reinoso-Suarez (1961) cat brain atlas, standard steel screws welded to a conductor cable for EEG recording were placed bilaterally on the skull over the frontal somato-motor cortex (2 mm rostral to Bregma and 10 mm from the midline) and the occipital primary visual cortex (21 mm caudal to Bregma and 2 mm from the midline). A reference screw electrode was implanted in the midline over the frontal sinus. Silver wires (380 μm diameter) were attached subcutaneously to both supraorbital ridges to record the electro-oculogram (EOG). Stainless steel wires were implanted in the neck muscles for electromyogram (EMG) recordings. Twisted stainless steel wires were placed subcortically with stereotaxic methods to record PGO activity in the lateral geniculate nucleus (LGN, AP, +7; V, +14; L, ±10) and hippocampal EEG in the CA1 region (AP, +3; V, +16; L, ±6). We also implanted a stainless steel guide tube or cannula (20 gauge) provided with a stylet. The cannula was inserted at a 32° angle to the coronal plane through the posterior fossa and, its tip, aimed at the PLCα nucleus (AP, −2/−3; V, 7; L, ±2), was left 4 mm above the target. All wires were affixed to an Amphenol strip connector, which, together with the cannula, was anchored to the skull with acrylic cement. After surgery, animals were given antibiotic and analgesic treatment for 5 days.

### Polygraphic Recordings and Drug Administration

After 8–10 days of recovery, each cat was placed in a soundproof ventilated recording chamber with constant temperature (22 ± 2°C) and dark/light cycle (12/12 h lights on at 7:00 h) for a 3–5 day habituation period. Food and water were supplied *ad libitum*. The animals were video recorded while they were in the soundproof chamber. Thereafter, 6 h polygraphic-video recordings starting at 10:30–11:00 h were made at 1 week intervals on a randomized schedule after PLCα unilateral microinjections of sterile saline (for baseline o control recordings) and after two doses of carbachol (0.01 and 0.1 M, Carbamylcholine chloride; Sigma), a long lasting cholinergic agonist.

All of the microinjections were performed with the cats awake and gently restrained, introducing the needle of a 0.5 μL Hamilton syringe through the cannula. The tip of the syringe protruded 4 mm from the end of the cannula and 20–30 nL of saline/carbachol were delivered. The syringe was left in place for 1 min before its removal in order to avoid leakage of the microinjected solutions along the needle track. Afterward, cats were placed in the soundproof chamber to immediately begin the polygraphic-video recordings.

The first 3 h of the polygraphic recordings were simultaneously digitized, filtered at 0.3–30 Hz and fed into a computer at a sampling frequency of 200 Hz for offline analysis. Spike2-CED software (Cambridge Electronic Design, Cambridge, United Kingdom) was used.

### Histology

At the end of the experiments the animals were given an overdose of Pentobarbital (100 mg/kg intraperitoneal) and perfused transcardially with saline, 10% formalin and increasing concentrations of sucrose (5, 10, and 20%). Frozen coronal sections of the brain were serially cut at 40–50 μm thicknesses, stained (Nissl) and examined. The site of the microinjections as well as the position of the subcortical electrodes were respectively identified in all the cats by locating the tip of the needle tract or the tip of the electrodes. The cat brain atlas of Reinoso-Suarez (1961) was used for the analysis.

### Analysis of the Polygraphic Recordings

**FIGURE 1:**
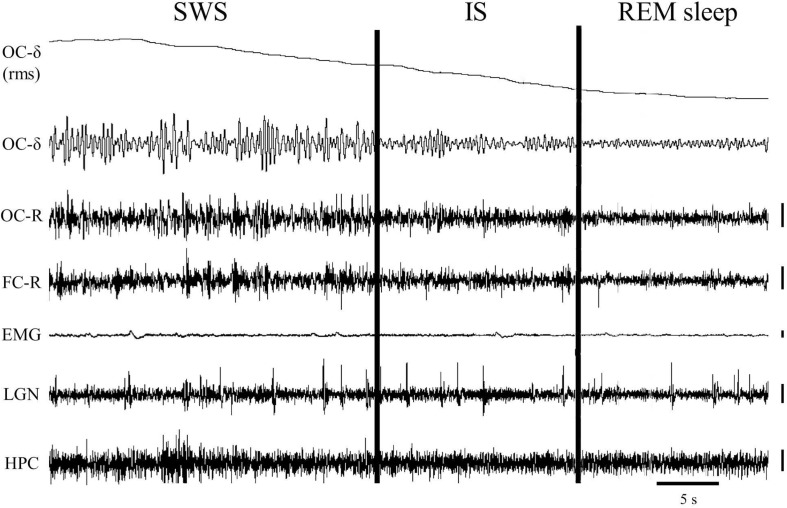
One-minute epoch of a polygraphic recording showing the transition from slow-wave sleep (SWS) to REM sleep. The vertical cursors delimit the IS interval selected in this particular animal. Notice the gradual decrease of the delta band voltage in the occipital cortex throughout the IS. Vertical calibration: 100 μV. OC-δ (rms), root mean square of the delta band signal from the occipital cortex; OC-δ, isolated delta band (1.5–3.5 Hz) from the occipital cortex; OC-R, occipital cortex-reference electrode; FC-R, frontal cortex-reference electrode; EMG, electromyogram; LGN, lateral geniculate nucleus; HPC, hippocampus.

Using polygraphic patterns for the physiological SWC stages in the cat (Ursin and Sterman, 1981), one episode containing the last minute of SWS with PGO activity together with the first minute of REM sleep was selected from baseline-saline recordings to detect and characterize IS (*n* = 10 cats). In these samples the occipital cortex δ band was isolated using the Spike2-CED software. For that, we used a 4^*th*^ order band-pass digital filter of the “Infinity Impulse Response” type (IIR) and “Butterworth” model, which allowed us to generate a new signal that only contained frequencies between 1.5 and 3.5 Hz. From the generated signal, its root mean square was calculated using a time constant of 0.05 s, and it was smoothed with the “smooth” filter using a time constant of 20 s. This made it possible to obtain a practically linear signal that represented the amplitude of the occipital cortex δ band over time (see section “Characterization of the Intermediate State” and Figure 1). This signal showed a progressive decline in the last seconds of SWS that reached minimum amplitude once EEG desynchronization of REM sleep was established. The interval with the highest slope values (see Figure 1) was taken into account in each cat to assess it as the IS. To determine whether or not these intervals actually corresponded to the IS, their bioelectric characteristics were compared with those of the SWS and REM sleep intervals of the same duration immediately before and after them.

After carbachol delivery in the PLCα nucleus, wakefulness with muscle atonia (Wa) and EEG synchronization with PGO waves (SPGO) were the main states observed. Latency to onset and time spent in these or the other stages of the SWC were quantified for the two first hours of recording. When REM sleep did not appear in those 2 h, the analysis continued until finding the first episode of REM sleep after PLCα carbachol microinjection. Additionally, for further off line analyses, the selected episodes of SWS and REM sleep on the baseline recordings and three 1-min SPGO epochs from the first one-and-a-half hour of experimental carbachol recordings (taken at 20 min intervals) were taken into account.

In the different polygraphic recording samples, the Spike2-CED software was used to obtain power spectra (frequency range: 0–20 Hz; resolution: 0.39 Hz) for the neocortical and hippocampal EEG from the selected intervals. The power values of EEG bands δ (0–3.5 Hz), θ (3.5–7.8 Hz), α (7.8–14 Hz), and β (14–20 Hz) were normalized in percentages of total power. In order to avoid signal contamination produced by ocular movements, the 0–1.5 Hz interval on the δ band was discarded in all cases. Moreover, PGO waves were quantified and the EMG signal was visually analyzed.

### Statistical Analysis

The Statview statistical package was used for the analysis. One-way ANOVAs for repeated measures were carried out to compare the relative values of the δ, θ, α, and β bands between: (1), IS and same length intervals of the previous SWS and the following REM sleep in control recordings; (2), 1-min periods of the three SPGO samples obtained after the low dose of carbachol in PLCα and both SWS and REM sleep of control experiments; and (3), the three SPGO samples obtained after low-dose carbachol and IS of control recordings. PGO waves were quantified and compared between the different states. In the cases in which the ANOVAs showed significant values, *post hoc* comparisons between value pairs were carried out applying Fisher’s least difference test. Statistical significance was set at *p* ≤ 0.05.

## Results

### Characterization of the Intermediate State

**FIGURE 2:**
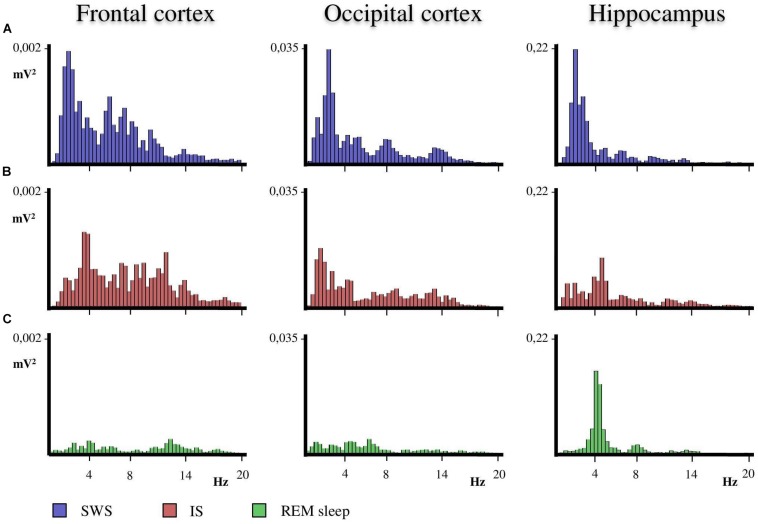
Representative power spectra obtained from frontal, occipital and hippocampal EEG recordings of one animal in the SWS-REM sleep transition. **(A)** The SWS prior to the progressive decline in the delta band interval that occurs before REM sleep onset. **(B)** The progressive decline in delta band interval during IS. **(C)** REM sleep just after IS. The duration of the IS interval was used to obtain the power spectra of the previous SWS and following REM sleep seconds. Thus, the three intervals had the same duration so as to obtain the corresponding power spectra. Calibration of Y axis is for all EEG frequencies (1.5–20 Hz) referenced to the values of SWS as the fully synchronized EEG state.

**FIGURE 3:**
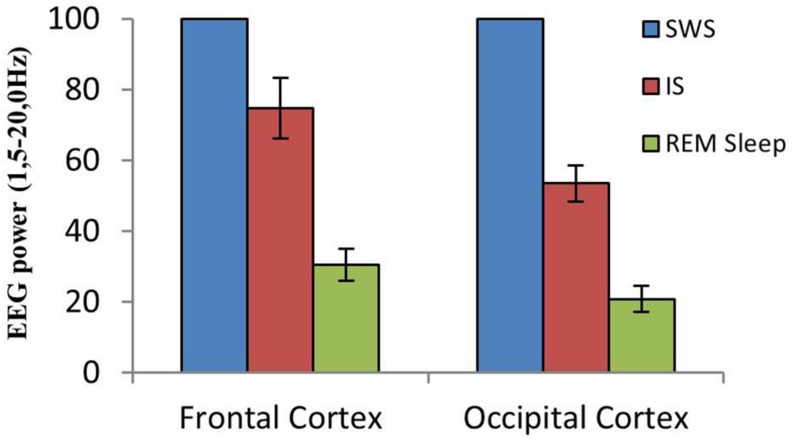
Cortical EEG synchronization/desynchronization at the SWS to REM sleep transition. Mean and standard error of EEG power values (1.5–20.0 Hz) in frontal and occipital cortices from equivalent-duration intervals of SWS, IS and REM sleep (*n* = 10 cats). Mean SWS EEG power was assumed to be 100%.

**FIGURE 4:**
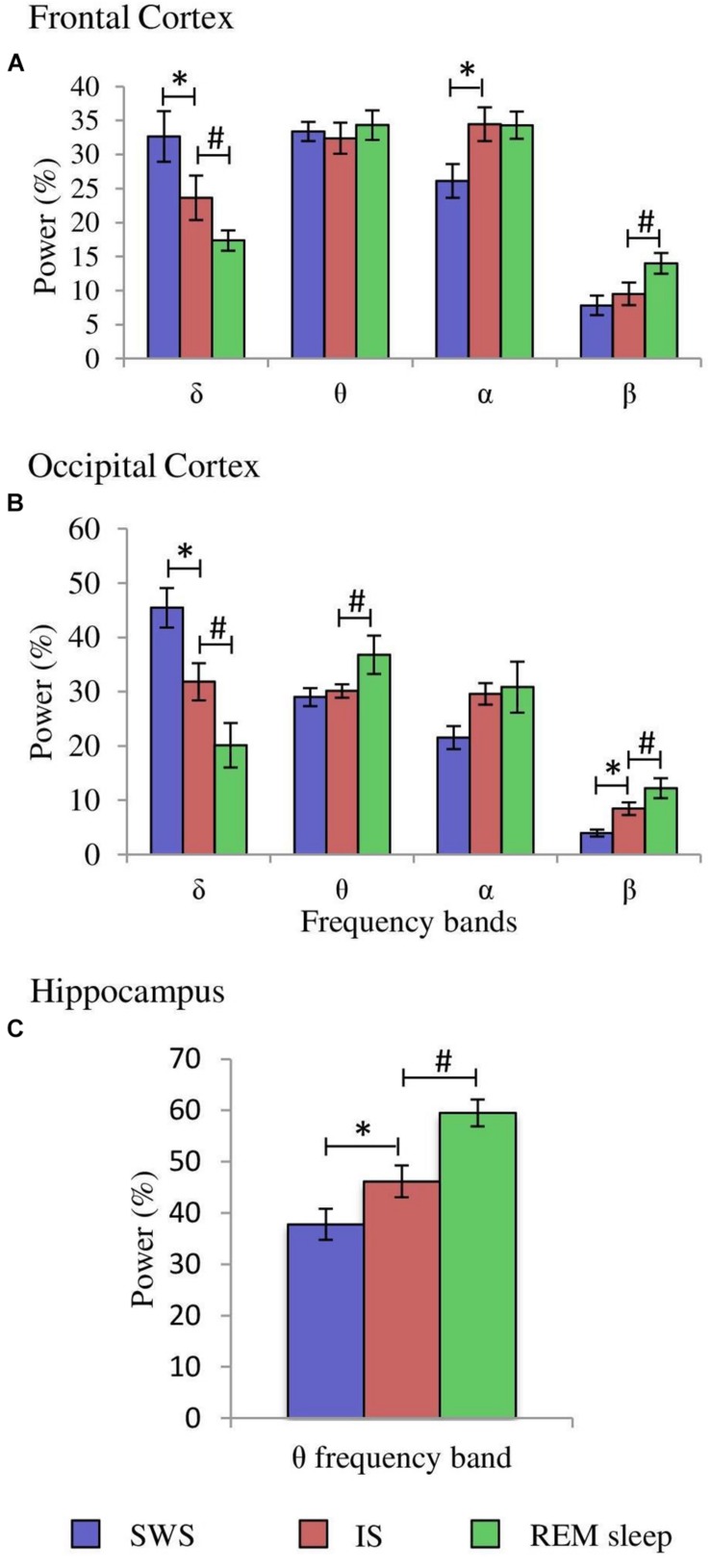
Comparisons of normalized power values for the different frequency bands between the IS and SWS as well as REM sleep. Each bar represents the average percentage value and the standard error of each EEG band relative to the total power (1.5–20 Hz) in: **(A)** frontal cortex, **(B)** occipital cortex, and **(C)** hippocampus (*n* = 10 cats). ^*^Statistically significant differences between IS and SWS. ^#^Statistically significant differences between IS and REM sleep. *Post hoc* analyses (Fisher’s test, *p* = 0.05).

A close examination of the 1-min SWS with PGO activity that preceded REM sleep indicated that the EEG of the frontal and occipital cortices did not abruptly change from a synchronized SWS pattern to a fully desynchronized one in the REM sleep episodes. The EEG in both the frontal and occipital cortices showed a noticeable tendency to desynchronization in the last few seconds of SWS (see Figure 1). This fact was well-documented when a root mean square analysis was applied to determine the voltage variation in the occipital cortex δ band in the 1-min SWS with PGOs and in the succeeding first minute of REM sleep samples. The signal showed a high and quite stable amplitude during SWS except for the last seconds, when a progressive decline took place before reaching the minimum amplitude once desynchronized EEG of REM sleep was well-established (Figure 1). The duration of these intervals showed some variability in the different cats, presenting a mean length of 14.6 ± 1.3 s (*n* = 10 cats). Also, as reflected by the frontal and occipital cortical power spectra (1.5–20 Hz) (Figure 2), the interval presenting the progressive decline in δ band power (Figure 2B) had an intermediate level of cortical synchronization (for the frontal and the occipital cortices) between the maximal EEG synchronization present in the preceding seconds of SWS (Figure 2A) and the minimal EEG synchronization in the following seconds that showed all the REM sleep characteristics (Figure 2C). Quantitative analysis comparing the total power values (1.5–20 Hz) of the frontal and occipital power spectra in these three intervals in the 10 cats (that is, between the interval showing the highest δ voltage variation and the two other accompanying intervals before and after it) confirmed the intermediate value of EEG synchronization in the former interval in comparison with the other two (Figure 3). Therefore, in terms of general cortical EEG synchronization/desynchronization, the interval showing the highest voltage variation for the δ band in the occipital cortex represented an intermediate state (IS) between the previous seconds, which had the typical EEG characteristics of SWS, and the subsequent ones, which had unquestionable EEG REM sleep features. Hippocampal θ in the three intervals also confirmed the intermediate character of these IS intervals in the 10 cats (Figures 2, 4C).

After having determined the duration of the IS in each of the 10 cats, further quantitative analyses were carried out. The δ, θ, α, and β bands normalized power values (as percentages of the total 1.5–20.0 Hz power) of the cortical EEG and hippocampal θ were compared between IS and the intervals of the same duration of the stages that preceded (SWS) and followed (REM sleep) it. Frontal cortex one-way ANOVAs for repeated measures showed significant differences for the δ (*F*_2,29_ = 20.433, *p* = 0.0001), α (*F*_2.29_ = 4.589, *p* = 0.0245), and β bands (*F*_2,29_ = 4.985, *p* = 0.0189). However, the differences for the θ band (*F*_2,29_ = 0.234, *p* = 0.794) were not significant. *Post hoc* comparisons demonstrated that in frontal cortex (Figure 4A, frontal cortex): (1), δ band power was significantly lower during IS than in SWS, but IS had higher values compared with REM sleep; (2), the power of the α band was significantly increased in IS versus SWS; and (3), the β band power was significantly increased in REM sleep with respect to IS. Concerning the occipital cortex, significant changes occurred in the δ (*F*_2,29_ = 55.448, *p* = 0.0001), θ (*F*_2,29_ = 3.716, *p* = 0.0446) and β bands (*F*_2,29_ = 25.08, *p* = 0.0001), but the differences for the α band did not reach significant values (*F*_2,29_ = 2.452, *p* = 0.1144). Pairwise comparisons (Figure 4B, occipital cortex) showed that: (1), the intermediate power values for the δ and β bands during IS reached statistically significant differences compared to both SWS and REM sleep; and (2), the θ band power was significantly higher during REM sleep than during IS. Regarding the hippocampal θ, there were significant differences (*F*_2,29_ = 17.418, *p* = 0.0001) between IS and both SWS and REM sleep (Figure 4C, hippocampus). The intermediate value of the hippocampal θ power was significantly different in comparison with the other two states. All these results further support the individuality of the IS with respect to SWS and REM sleep.

Regarding the PGO wave analyses in the 10 cats, isolated and double PGO waves constituted an average of 95 and 97.5% of the total PGO waves in the last seconds of SWS prior to REM sleep and IS respectively, but were only 67.5% of the PGO waves in the first seconds of REM sleep. The remaining percentage in each case corresponded to PGO waves firing in clusters (≥3 PGO waves), something which occurred only occasionally during SWS and IS. The isolated PGO waves remained unchanged during SWS (60.5 ± 7.6%) and IS (62.4 ± 7.9%), but were significantly less frequent in REM sleep (*F*_2,29_ = 3.585, *p* = 0.04). However, the proportions of double PGO waves did not show significant differences (*F*_2,29_ = 0.264, *p* = 0.7709) between SWS (34.5 ± 8.4%), IS (35.1 ± 7.6%), and REM sleep (26.4 ± 6.1%). Finally, although the EMG signal was not quantified, examining the polygraphic recordings of the animals indicated that the complete loss of muscle tone occurred either at the end of the IS or at the beginning of REM sleep.

### Carbachol Experiments

**FIGURE 5:**
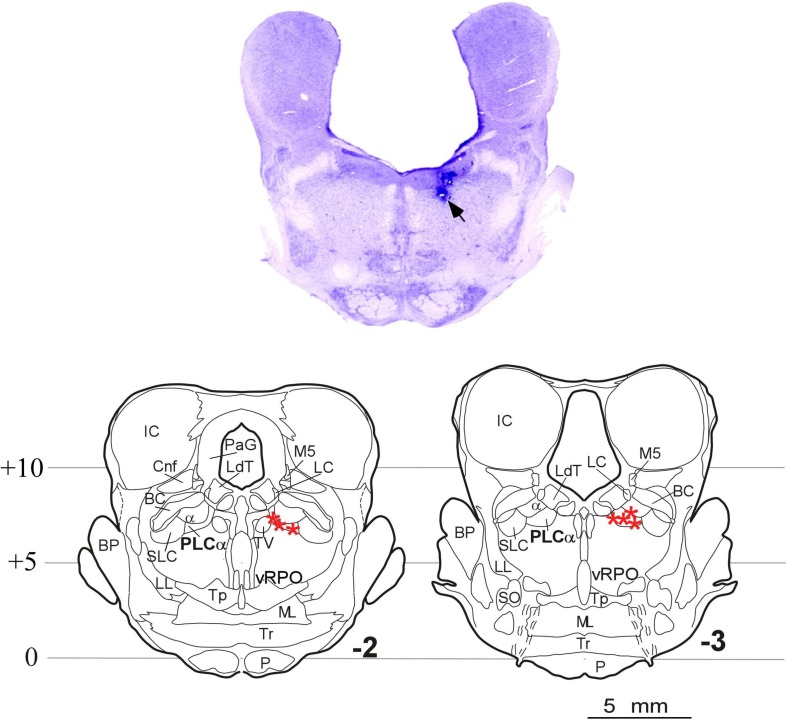
Carbachol microinjection locations. **(Upper)** Photomicrograph of a brainstem coronal section stained with Nissl method from an animal with the two doses of carbachol and control-saline microinjections in the rostrodorsal pontine tegmentum at the level of the PLCα. The arrow indicates the site of the microinjections. **(Lower)** Coronal planes –2 and –3 of the Reinoso-Suarez (1961) atlas showing the location of the microinjection sites in the seven animals in which the PLCα nucleus was reached (stars). α, locus coeruleus alpha; BC, brachium conjunctivum; BP, brachium pontis; Cnf, cuneiform nucleus; IC, inferior colliculus; LC, locus coeruleus proper; LdT, laterodorsal tegmental nucleus; LL, lateral lemniscus; ML, medial lemniscus; M5, mesencephalic trigeminal tract; P, pyramidal tract; PLCα, perilocus coeruleus alpha; PaG, periaqueductal gray; vRPO, ventral part of the oral pontine reticular nucleus; SLC, locus subcoeruleus; SO, superior olivary nucleus; Tp, tegmental pontine nucleus; Tr, trapezoid body; TV, ventral tegmental nucleus.

Histological analyses indicated that seven of the 10 cats used in this study received microinjections situated in the PLCα; the remaining three cats had received the injections outside the target region, so they were excluded from the carbachol studies. Figure 5 shows the microphotograph illustrating the location of the microinjections in one animal, and the microinjection sites of the seven animals in coronal drawings using the cat brainstem from the Reinoso-Suarez (1961) atlas. Note that in all cases the microinjections in the rostrodorsal pontine tegmentum were located at the level of the PLCα.

After microinjecting small volumes of the long lasting cholinergic agonist carbachol in the PLCα, all the cats showed, at short latency, two states that did not polygraphically fit the physiological patterns of the cat SWC (Table 1). These two states were: (1) Wa, a state of wakefulness without muscle tone (that is, with muscle atonia), and (2) the state that we have called SPGO, in which muscle atonia and PGO activity were associated with EEG synchronization. There were dose-response effects in terms of the latency to onset and time spent in these states; the high carbachol dose (0.1 M) promoted a shorter latency and greater proportion of Wa, while the low dose (0.01 M) promoted the SPGO state (Table 1). Also, the administration of either carbachol dose in the PLCα practically abolished the physiological stage of SWS and caused wakefulness with muscle tone to appear with long latencies and in low proportions (Table 1). Concerning REM sleep, except in one animal in which a REM sleep episode was observed with a latency of 18 min, REM appeared after a long latency, more than an hour after carbachol delivery (Table 1).

**TABLE 1:** Latency to onset and time spent (mean ± standard error) in the different sleep-wakefulness states observed in the first 2 h after unilateral carbachol miroinjections in the rostrodorsal pontine tegmentum at the level of the PLCα (*n* = 7 cats).

**Carbachol dose (M)**	**State**	**Latency (min)**	**Time spent (min)**	
			**Hour 1**	**Hour 2**
0.1	Wa	1.6 ± 0.6	31.0 ± 8.8	20.3 ± 10.8
	SPGO	15.8 ± 9.1	20.3 ± 8.6	14.5 ± 7.7
	Wt	40.8 ± 22.7	8.8 ± 4.7	23 ± 11.2
	REM	>90	0	2.3 ± 2.3
0.01	Wa	24.8 ± 11.6	6.2 ± 1.8	15.5 ± 11
	SPGO	3.9 ± 1.76	45.2 ± 3.8	37.8 ± 9.1
	Wt	40.8 ± 12.6	8.6 ± 4.2	4 ± 2.1
	REM	>110	0	2.8 ± 2.8

*SPGO, EEG synchronization with pontogeniculooccipital activity; REM, REM sleep; Wa, wakefulness with muscle atonia; Wt, wakefulness with muscle tone.*

### Matching Carbachol-Induced States With the Physiological Stages of the Sleep-Wakefulness Cycle

#### Comparisons With SWS and REM Sleep

**FIGURE 6:**
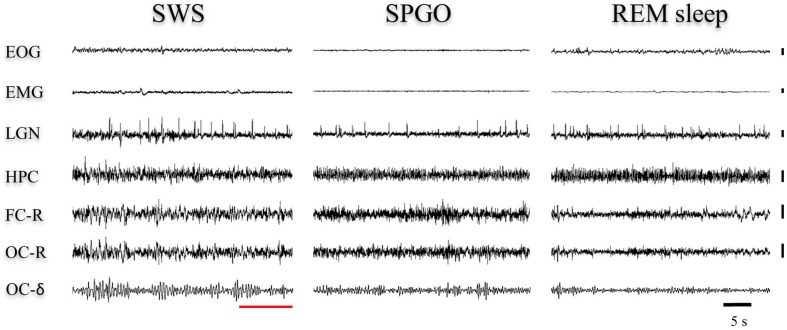
Representative 40-s epochs of polygraphic recordings in one animal from **(left)** the end of SWS before REM sleep, **(middle)** the SPGO state, and **(right)** the beginning of a REM sleep episode. The red line shows how, within the SWS episode, the last seconds display EEG voltage changes that would correspond to the IS. These voltage features are visually similar to cortical and hippocampal leads in the SPGO state. Vertical calibration: 100 μV. EOG, electrooculogram; EMG, electromyogram; LGN, lateral geniculate nucleus; HPC, hippocampus; FC-R, frontal cortex-reference; OC-R, occipital cortex-reference. OC-δ, isolated delta band (1.5–3.5 Hz) from the occipital cortex.

**FIGURE 7:**
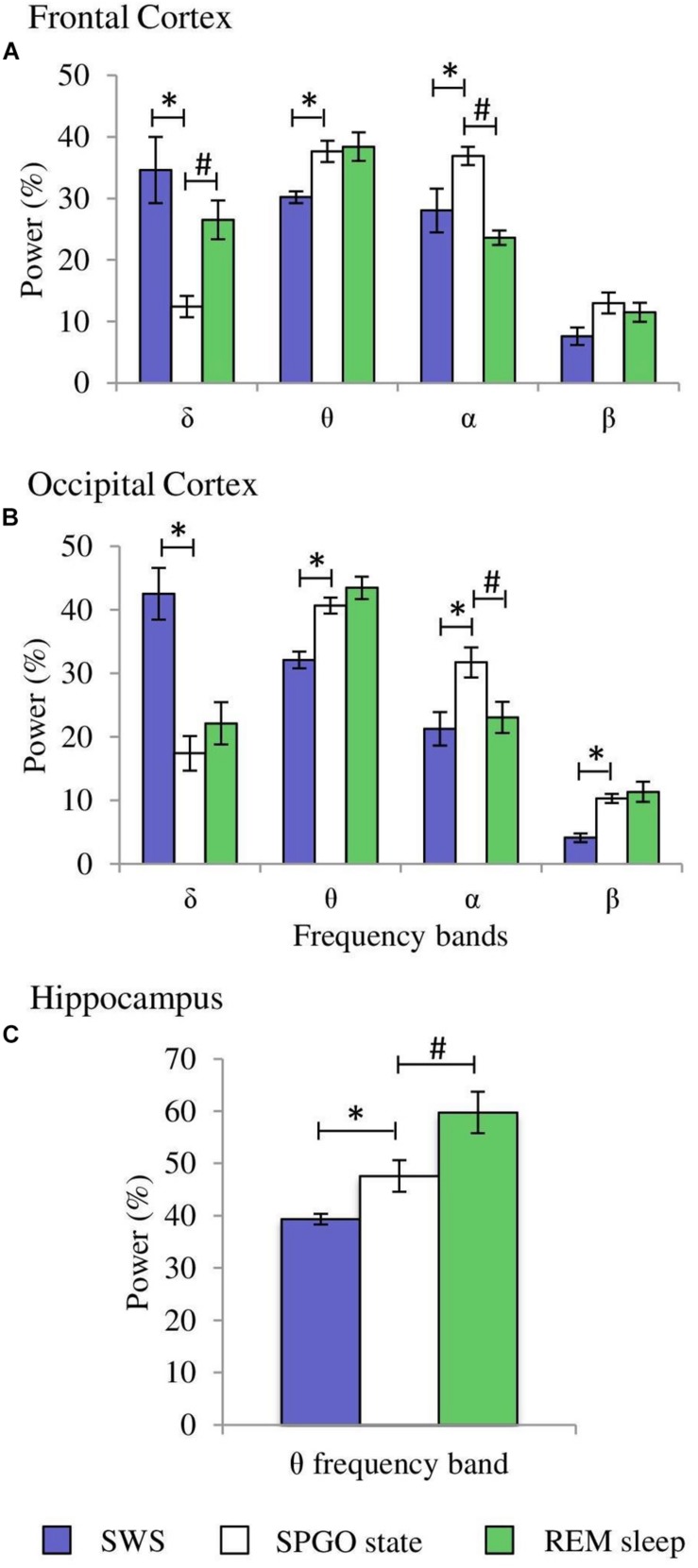
Comparisons of normalized power values for the different EEG frequency bands between the SPGO state, SWS and REM sleep. Each bar represents the average percentage value and the standard error of each EEG band relative to the total power (1.5–20 Hz) from 1-min epochs in the different states. **(A)** Frontal cortex, **(B)** occipital cortex, and **(C)** hippocampus (*n* = 7 cats). Values for the SPGO state are the mean ± standard error from the three successive 1-min epochs (20 min apart) obtained from each animal after 20–30 nl 0.01 M carbachol in the PLCα. ^*^Statistically significant differences between the SPGO state and SWS. ^#^Statistically significant differences between SPGO state and REM sleep. *Post hoc* analyses (Fisher’s test, *p* = 0.05).

The Wa state is comparable to the state of cataplexy (Reinoso-Suárez et al., 1994; Moreno-Balandrán et al., 2008; Torterolo et al., 2015, 2016) in which the electroencephalographic characteristics are common to those of the W stage, but there is a loss of muscle tone. However, the SPGO state is more complex since it constitutes a behavioral sleep state with a dissociated polygraphic pattern in which characteristic manifestations of SWS (EEG synchronization) and REM sleep (θ rhythm in the hippocampus, PGO waves in the LGN and muscle tone absence) coexist (Figure 6). Therefore, the three 1-min samples of the SPGO state taken sequentially from the first hour-and-a-half of experimental recordings were compared with the last minute of SWS with PGOs (which included the few seconds of IS intervals) prior to REM sleep, and with the first minute of REM sleep. One-way ANOVAs for repeated measures for the normalized power for the different frequency bands of the cortical EEG and for the hippocampal θ were carried out comparing the values of the SPGO state with those of the SWS prior to REM sleep and the subsequent REM sleep. For the frontal cortex, the analyses indicated that there were significant differences for the δ (*F*_4,34_ = 12.87, *p* = 0.0001), θ (*F*_4,34_ = 4.576, *p* = 0.0069), and α bands (*F*_4,34_ = 9.89, *p* = 0.0001). However, the differences for the β band were not significant (*F*_4,34_ = 2.659, *p* = 0.0574). *Post hoc* comparisons indicated that the different EEG bands did not significantly differ between the three samples of SPGO activity taken sequentially after carbachol administration (Supplementary Table S1). Instead, for the frontal cortex (Figure 7): (1), the power of the δ band was significantly lower in the SPGO state than in SWS or even in REM sleep; (2), the power of the θ band was significantly increased during the SPGO state compared with SWS; and (3), the α band power was significantly higher during the SPGO state than in SWS and REM sleep. For the occipital cortex there were significant differences between the states for all the frequency bands: δ (*F*_4,34_ = 22.463, *p* = 0.0001); θ (*F*_4,34_ = 8.138, *p* = 0.0003); α (*F*_4,34_ = 7.239, *p* = 0.0006), and β (*F*_4,34_ = 7.899, *p* = 0.0003). Pairwise comparisons again showed that the different EEG bands did not significantly differ between the three samples of SPGO activity (see Supplementary Table S1), but, in the occipital cortex (Figure 7B, occipital cortex): (1), the δ band power was significantly lower in the SPGO state than during the SWS; (2), the power of the θ and β bands was significantly higher during the SPGO state than in SWS; and (3), the α band power was significantly higher during the SPGO state than in either SWS or REM sleep. Concerning the power of hippocampal θ, significant differences occurred (*F*_4,34_ = 12.705, *p* = 0.0001), but, according to *post hoc* comparisons, there were again no significant changes between the three SPGO samples (see Supplementary Table S1). Instead, hippocampal θ band power during SPGO was significantly higher than in SWS but significantly lower than in REM sleep (Figure 7C). It should be noted that as no differences were found between the three samples of the SPGO state in any case, Figure 7 groups the values of the three SPGO samples for the comparisons of this state with SWS and REM sleep. Summing up, these results show that SPGO presented: (1), low power values for the δ band in frontal and occipital cortices with respect to SWS; (2), higher power values for α activity in both cortices than in SWS and REM sleep; and (3), intermediate values for the hippocampal θ rhythm, higher than in SWS but lower than in REM sleep. Therefore, these results demonstrate the lack of an analogy between this state and SWS and REM sleep, as well as suggesting that the SPGO state could be equivalent to the IS.

#### Comparisons With IS

**FIGURE 8:**
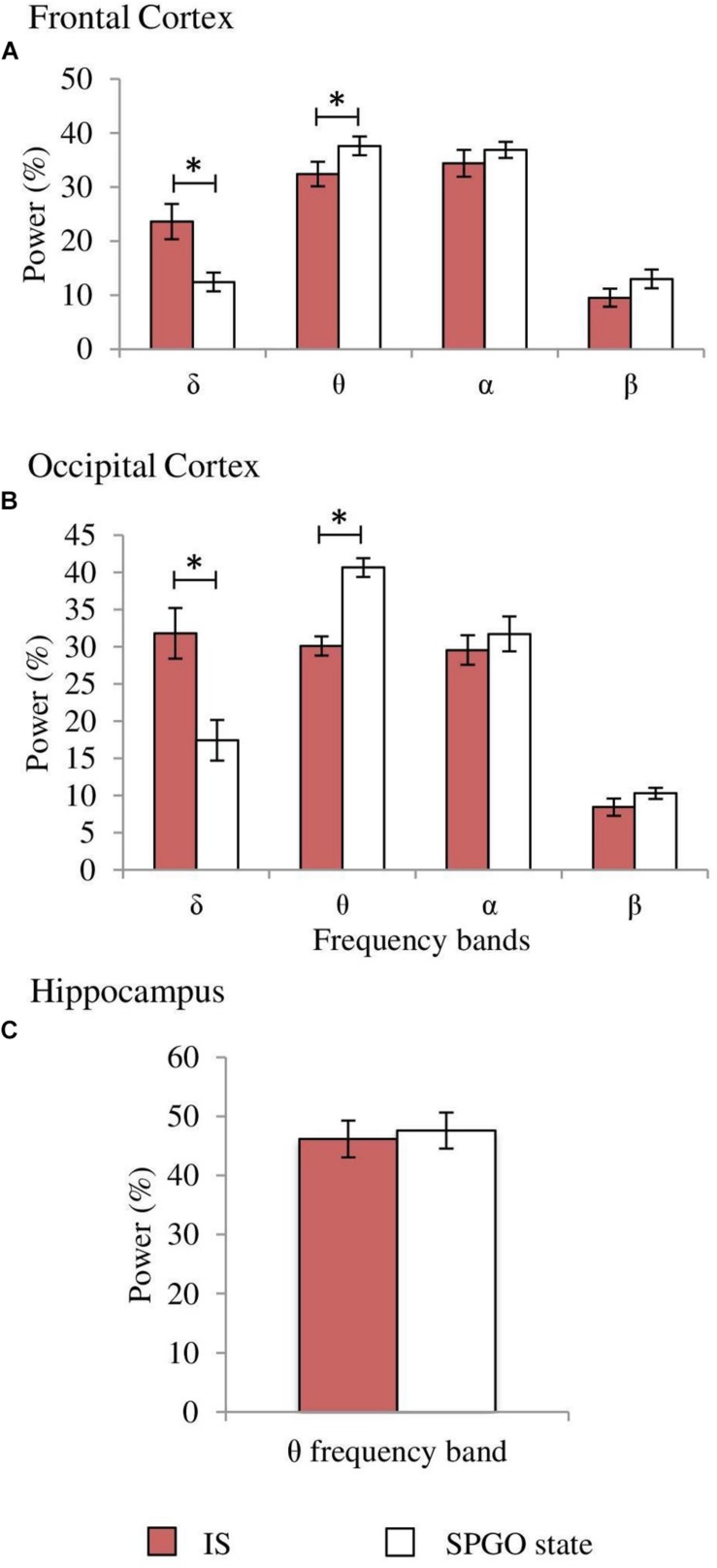
Comparison of normalized power values of the different frequency bands between the IS and the SPGO state. Each bar represents the average percentage value and the standard error of each EEG band relative to the total power (1.5–20 Hz) in **(A)** frontal cortex, **(B)** occipital cortex, and **(C)** hippocampus (*n* = 7 cats). Values for the SPGO state are the mean ± standard error from the three successive 1-min epochs (20 min apart) obtained from each animal after 20–30 nl 0.01 M carbachol in the PLCα. ^*^Statistically significant differences between IS and SPGO. *Post hoc* analyses (Fisher’s test, *p* = 0.05).

One-way ANOVAs for repeated measures comparing the values of the SPGO state and the IS for the frontal cortex indicated that there were significant differences for the δ (*F*_3,27_ = 7.765, *p* = 0.0016) and θ bands (*F*_3,27_ = 3.867, *p* = 0.0269), but not for the α (*F*_3,27_ = 0.711, *p* = 0.5579) and β bands (*F*_3,27_ = 1.211, *p* = 0.3343). *Post hoc* comparisons (Figure 8A, frontal cortex) showed that the power of the δ band was significantly lower in the SPGO state than in IS, and that the power of the θ band was significantly higher during the SPGO state than in IS. In relation to the occipital cortex, as well as the frontal cortex, there were significant differences between the SPGO state and the IS for the δ (*F*_3,27_ = 8.391, *p* = 0.0011) and θ bands (*F*_3,27_ = 10.777, *p* = 0.0003), but there were no significant differences with the α (*F*_3,27_ = 0.83, *p* = 0.4947) and β bands (*F*_3,27_ = 0.901, *p* = 0.4601). *Post hoc* comparisons (Figure 8B, occipital cortex) indicated that δ band power was significantly lower in the SPGO state than in the IS, whilst θ band power was significantly higher during the SPGO state than in the IS, again like occurring in the frontal cortex. Finally, comparing the hippocampal θ band powers between the two states (Figure 8C) indicated that there were no significant differences between them. Since differences between the three samples of the SPGO state were not found in any case (see Supplementary Table S1), Figure 8 groups the values of the different SPGO samples for the purpose of comparing this state with the IS, as was done in all above comparisons.

In relation with PGO waves, the proportions of isolated PGOs in the seven animals were similar in the three SPGO state samples (mean percentages: 58.6 ± 5.4, 60.7 ± 4.1, and 59.9 ± 5.8%) and IS (59.1 ± 9.7%) without significant differences between them (*F*_3,27_ = 0.022, *p* = 0.9953). The proportions of double PGO waves were also unchanged (*F*_3,27_ = 0.39, *p* = 0.7617) in the three SPGO samples (mean percentages: 34.7 ± 4.6, 28.8 ± 4.6, and 33.6 ± 5.6%) as well as being similar to IS (37.3 ± 9.3%). Thus, PGO data gives further indications of strong similarities between the SPGO state induced by cholinergic stimulation of the PLCα region and physiological IS.

## Discussion

Currently there is great interest in studying how the transition to REM sleep occurs, specifically an interest in characterizing the IS and understanding its neural mechanisms, since the key to some pathologies like narcolepsy may lie in this transition (Sorensen et al., 2013). The cat is a good experimental model for studying the neurobiological bases of the transition to REM sleep since a large amount of our knowledge about the mechanisms of the SWC has been accumulated in this species and, additionally, the electrographic manifestations of the sleep phases in the cat, like PGO activity a landmark of REM sleep but also of the IS as reported in the present work- is both easier to record and better characterized in cats than in rodents. As far as we know, only one work has described the characteristics of IS in the cat (Gottesmann et al., 1984), and those characteristics, especially in regards to duration, are quite different from the IS characteristics described in recent studies in the rat (Sánchez-López et al., 2018). To reevaluate IS in cats we have first tried to identify its location and duration based on two widely accepted criteria from the literature associated to REM sleep onset: EEG desynchronization; and the decrease of δ band amplitude in the occipital cortex, where this activity is particularly patent during the deep SWS with PGOs that precedes REM sleep in the cat (Ursin and Sterman, 1981). These criteria make it easy to detect, as shown in Figure 1, the existence of a short yet very dynamic period, although with slight differences in each animal, that lies between SWS and REM sleep. This period is not abrupt, but has a progressive presentation as indicated by the slope reflecting the amplitude decrease in the occipital cortex δ band. Accordingly, the power spectra for this period (Figures 2, 3) had values that were intermediate between those of the preceding and following intervals with the same duration. Therefore, these observations would ensure that the chosen period was a transition state corresponding to the IS, since its cortical and hippocampal EEG was qualitatively and quantitatively different from that of both the preceding SWS and the subsequent REM sleep.

According to the above criteria, our results show that, in contrast to previous work done in cats in relation with this topic (Gottesmann et al., 1984), the IS in the cat has an average duration of 14.6 ± 1.3 s. The analyses of the relative powers of the different cortical EEG bands indicate that the IS in cats is characterized by: (1), a significant decrease in amplitude of the δ band in the cortices with respect to the previous SWS, to a value that is even lower in the succeeding REM sleep; (2), a significant increase in amplitude of the α band in the frontal cortex compared with the previous SWS, which would correspond to the large amplitude sleep spindles previously described during the IS (Gottesmann et al., 1984), an activity that decreases once REM sleep has started; (3), a significant increase in hippocampal θ in relation with the previous SWS, although the hippocampal θ does not become as powerful and rhythmic during IS as during REM sleep. In contrast, PGOs during IS did not differ significantly from those in the preceding SWS; isolated and double PGO waves occurred in both cases and in the same proportions. PGOs are considered to be the physiological signals that trigger REM sleep (Callaway et al., 1987). Our results support this point of view, and they add the probability that PGO activity is also necessary to trigger IS since PGOs are already present with the full EEG characteristics of SWS before IS appears. Concerning muscle tone, the IS presented a decrease of muscle tone with respect to the previous SWS, and that tone was completely lost either by the end of the IS or at the beginning of REM sleep. Finally, it is particularly noteworthy that our results indicate that, as in rats (Sánchez-López et al., 2018), the transition from SWS to REM sleep is not an abrupt phenomenon as occurs with the transition from SWS to wakefulness when the reticular formation is stimulated (Moruzzi and Magoun, 1949). Entrance into REM sleep occurs gradually over an intermediate stage, the IS. This implies the need to reconsider the transition models from SWS to REM sleep with flip-flop characteristics, that is, with sharp transitions from one state to another (Lu et al., 2006).

In relation with the carbachol experiments, as our results show, the cholinergic stimulation of the PLCα nucleus produced complete disturbance of the SWC, totally abolishing some stages of the cycle such as SWS, while considerably increasing the latency of others, like REM sleep. Instead of those, two new states appeared: W with atonia (Wa), mainly after high carbachol doses, and a state called SPGO, which was more frequent after low doses. During Wa the cats had increased respiratory and heart rates and they also had intermittent recoveries of muscle tone, which confirmed that the animals were paralyzed but awake (Reinoso-Suárez et al., 1994; Moreno-Balandrán et al., 2008; Torterolo et al., 2015). Therefore, during Wa the animals showed a clear analogy with the state of cataplexy. In contrast, during SPGO the cats were behaviorally asleep but showed a dissociated polygraphic pattern (Baghdoyan et al., 1982) that did not present a complete analogy with any of the physiological stages of SWS or REM sleep.

Both carbachol-induced states presented a common characteristic, the absence of muscle tone typical of REM sleep, confirming that the PLCα is a fundamental region involved in the triggering of partial signs of REM sleep such as muscle atonia. This observation concurs with results from experiments with bilateral PLCα lesions in cats, in which REM sleep without muscle atonia was observed (Jouvet, 1979; Sastre and Jouvet, 1979). Similarly, SLD nucleus lesions in the rat produced REM sleep with muscle tone (Lu et al., 2006). These conclusions would support the role of the PLCα/SLD region for the triggering of muscle atonia during REM sleep through its descending projections to the inhibitory interneurons of the spinal cord that would inhibit motoneurons. The PLCα/SLD projections would have an intermediate station in the ventromedial medulla (Sakai, 1985) or reach the spinal cord interneurons directly (Lu et al., 2006). Another feature of REM sleep observed after cholinergic stimulation of the PLCα nucleus is the presence of PGO waves. It is well-known that the rostrodorsal pontine tegmentum contains structures that mediate PGO activity after carbachol administration (Datta et al., 1993, 1998). Our work demonstrates that the type of PGO waves that appear when the PLCα nucleus is cholinergically stimulated are isolated or double waves, just like the PGO patterns that precede REM sleep episodes and that are present in both the SWS and IS.

In our experiments, cholinergic stimulation of PLCα always produced muscle atonia, but this was not the case for PGO activity. After administering the high dose of carbachol we did not immediately observe PGO waves, contrary to the PGO activity observed in response to the low dose. The SLD nucleus in the rat holds several neuronal groups with differential responses to carbachol administration and some show a low bursting threshold similar to the firing of other pontine neurons known to be involved in PGO wave generation (Brown et al., 2006). The use of M2 acetylcholine muscarinic receptor antagonists blocks carbachol-induced PGOs (Datta et al., 1993). On the other hand, in cats, the inhibition of the M3 type acetylcholine muscarinic receptors in the PLCα nucleus had, as a consequence, the absence of muscle atonia during REM sleep (Sakai and Onoe, 1997). M2 receptor carbachol affinity is almost five times higher than that of the M3 receptor (Vanderheyden et al., 1990). Consequently, the absence of PGO activity after administering the high carbachol dose is probably mediated by some M2 receptor desensitization mechanism, such as receptor internalization, in order to maintain homeostasis in the neurons generating that activity. This would explain why PGO waves are not readily observed after the administration of the high carbachol dose, since they would not appear until sufficient time had elapsed for the extracellular concentration of the cholinergic agonist to be reduced.

Some authors have proposed that the PLCα is the responsible region for triggering complete REM sleep (Sakai, 1988; Vanni-Mercier et al., 1989; Boissard et al., 2002; Lu et al., 2006). However, according to our results, cholinergic stimulation of the PLCα nucleus does not produce complete REM sleep, only the appearance of some signs of REM sleep mixed with characteristics of other stages of the SWC. This agrees with previous works by our group, which found that the vRPO region was the only region in which the administration of low-volume microinjections with a wide range of carbachol doses produced REM sleep with all its manifestations (Reinoso-Suárez et al., 1994; Garzón et al., 1997, 1998; Moreno-Balandrán et al., 2008).

Returning to the SPGO state, it is not truly comparable to either SWS or REM sleep. However, it has a much better match with IS, since both states show, in comparison to SWS, increased α band amplitude in the frontal cortex, decreased δ band amplitude in both cortices, increased θ rhythm amplitude in the hippocampus, and decreased muscle tone, as well as presenting similar proportions of single and double PGO waves. The only significant differences observed between the SPGO state and IS were the lower δ band and higher θ band power in both cortices during the SPGO state. The decrease in δ power is one of the main EEG characteristics of IS in rats (Sánchez-López et al., 2018) and in cats as reported here. We think that the more pronounced δ decrease and its associated increase in θ power after carbachol in the PLCα could be explained by an excessive carbachol effect, one that would probably disappear at lower carbachol doses. Therefore, we believe that these differences do not suppose the rejection of an analogy between the IS and SPGO states.

Since stimulation of the PLCα nucleus with low carbachol doses induces SPGO, which constitutes a good expression of physiological IS, it seems that the PLCα region could be the organizing structure behind the transition from SWS (or NREM) sleep to REM sleep, therefore generating IS. It is striking that, in both the cat and the rat, α band voltage rises during IS. This could correlate with the fact that the entrance into REM sleep in humans does not occur from the N3 phase, an NREM sleep stage with predominance of δ waves, but always previously passes through the N2 phase that has predominant spindle α activity (Achermann and Borbély, 2011). Therefore, it seems that for REM sleep to occur, hyperpolarization levels in thalamo-cortical cells must vary beforehand since these cells are responsible for the EEG bioelectric manifestations during NREM sleep (De Andrés et al., 2011). Depending on the degree of hyperpolarization in thalamo-cortical neurons, typical SWS δ waves or sleep spindle α activity will be observed on the EEG (Nuñez et al., 1992). Accordingly, the electroencephalographic changes observed during IS, such as the voltage increase in the α band, will be mediated by the firing of reticular thalamic neurons transferred to the cortex through thalamic projection cells (Steriade et al., 1985, 1991). This effect could be carried out by neurons from the PLCα region, which has multiple direct connections with the various nuclei of the thalamus, including the reticular thalamic nucleus (Datta et al., 1998). Moreover, the hippocampal θ rhythm that characterizes REM sleep and that is enhanced during IS and SPGO compared to levels during SWS, as shown in the present work, is generated by GABAergic and cholinergic projections reaching the hippocampus from θ pacemaker neurons in the medial septum (Petsche et al., 1962; Gerashchenko et al., 2001; Fuller et al., 2007). However, brainstem structures are also involved in triggering this event, such as the excitatory projections reaching the medial septum from the precoeruleus region (Fuller et al., 2007) as well as from the nucleus incertus (Nuñez et al., 2006), which constitutes a relay station between the oral pontine reticular nucleus and the medial septum for hippocampal θ rhythm generation (Vertes, 1980; Nuñez et al., 1991). Therefore, it is likely that PLCα activation is also related to the increase of θ rhythmicity observed in the hippocampus during both IS and the carbachol-induced SPGO state. Finally, it is important to remember the reciprocal connections between the locus coeruleus complex and the vRPO region, which is responsible for the complete generation of REM sleep. The vRPO has strong connections with the thalamic nuclei, including reciprocal connections with the reticular nucleus (Reinoso-Suárez et al., 1994; Rodrigo-Angulo et al., 2008); thus the high α activity observed during IS could be blocked by the vRPO region thus giving way to the complete EEG desynchronization observed during REM sleep.

## Conclusion

The present results strongly indicate that the IS is an independent sleep stage, and confirm that the PLCα region, rather than a REM sleep triggering region, is a region that generates some partial signs of REM sleep, such as muscle atonia or PGO waves. Also, we present results that allow us to propose the PLCα nucleus as the structure organizing IS, which occurs in the transition from SWS to REM sleep.

## Data Availability

All datasets generated for this study are included in the manuscript and/or the Supplementary Files.

## Ethics Statement

All experiments were carried out in accordance with the European Community Council Directive (2010/63/UE) and approved by the Institutional Animal Care and Use Committee of the Universidad Autónoma of Madrid (Spain) and the competent regional government agency (PROEX 004/15).

## Author Contributions

CC-C scored and analyzed the polygraphic recordings, performed statistical analyses, and wrote the manuscript. MG performed the experiments and wrote the manuscript. IdA conceived and designed the experiments, supervised all aspects of the study, and wrote the manuscript.

## Conflict of Interest Statement

The authors declare that the research was conducted in the absence of any commercial or financial relationships that could be construed as a potential conflict of interest.
