# Supplementary material for: The Transition Between Slow-Wave Sleep and REM Sleep Constitutes an Independent Sleep Stage Organized by Cholinergic Mechanisms in the Rostrodorsal Pontine Tegmentum
Source: Front Neurosci. 2019 Jul 23;13:748. doi: 10.3389/fnins.2019.00748 (PMC6663996; doi:10.3389/fnins.2019.00748)
Supplement: Supplementary file 1 [file Table_1.DOCX]

Supplementary Material

**Table S1.** Mean power values and standard errors (in percentage of the total EEG power -1.5-20.0 Hz-) for the δ, θ, α and β EEG bands in the different one-minute samples of the SPGO state. Values obtained from the statistical comparisons (One-way ANOVAs for repeated measures) between the three SPGO samples are also provided. Abbreviations: FC, frontal cortex; OC, occipital cortex; HPC, hippocampus.

| **Brain structure** | **Frequency band** | **Power (percentage of total power)** | | | **Statistical comparisons** | |
| --- | --- | --- | --- | --- | --- | --- |
|  |  | **SPGO-1** | **SPGO-2** | **SPGO-3** | **F_(2,20)_** | **P value** |
| **FC** | **δ** | 13.2 ± 2.3 | 11.9 ± 1.6 | 12.2 ± 1.5 | 0.812 | 0.4667 |
|  | **θ** | 37.2 ± 1.6 | 37.9 ± 2.1 | 37.8 ± 2.3 | 0.99 | 0.9061 |
|  | **α** | 35.4 ± 1.8 | 38.1 ± 1.4 | 37.2 ± 2.0 | 1.601 | 0.2418 |
|  | **β** | 14.2 ± 2.1 | 12.0 ± 1.7 | 12.8 ± 1.7 | 1.825 | 0.2032 |
| **OC** | **δ** | 17.4 ± 3.2 | 18.5 ± 2.6 | 16.4 ± 1.9 | 1.342 | 0.298 |
|  | **θ** | 38.5 ± 1.2 | 43.1 ± 2.1 | 40.4 ± 2.0 | 1.992 | 0.179 |
|  | **α** | 33.4 ± 3.1 | 29.4 ± 2.4 | 32.4 ± 2.1 | 1.671 | 0.2289 |
|  | **β** | 11.2 ± 0.9 | 9.0 ± 0.8 | 10.7 ± 1.3 | 1.522 | 0.2576 |
| **HPC** | **θ** | 48.2 ± 2.5 | 47.5 ± 3.9 | 47.1 ± 3.5 | 0.113 | 0.8938 |
